# Mate locating and access behaviour of the parasitic pea crab, *Nepinnotheres novaezelandiae*, an important parasite of the mussel *Perna canaliculus*

**DOI:** 10.1051/parasite/2015013

**Published:** 2015-03-18

**Authors:** Oliver Trottier, Andrew G. Jeffs

**Affiliations:** 1 Leigh Marine Laboratory, Institute of Marine Science, University of Auckland Warkworth 0941 New Zealand

**Keywords:** Pea crab, *Perna canaliculus*, Host manipulation, Aquaculture, Pinnotherid, Greenshell

## Abstract

Pea crabs are globally ubiquitous symbionts in the marine environment that cause serious economic impact in the aquaculture production of several major bivalve species. However, little is known about their host-parasite interactions, especially the mating behaviour of these parasites that could prove useful for controlling their infestation in aquaculture. In this study, the mate location behaviour of male New Zealand pea crabs, *Nepinnotheres novaezelandiae* (Filhol, 1885), was observed when dwelling in its preferred host, the commercially important green-lipped mussel, *Perna canaliculus*. Given the cryptic behaviour of the male crabs, a novel trapping system was developed to determine whether male crabs would exit their mussel hosts in response to an upstream female crab. The presence of receptive female crabs placed upstream successfully attracted 60% of male crabs from their host over 24 h. Observations of the nocturnal mate-finding behaviour of male crabs were made in darkness using infrared video recordings. Males spent on average 49 min on empty hosts and never left a mussel containing a female conspecific once found, spending 200 min on average to gain entry to the mussel. Male crabs were often observed stroking the mantle edge of the mussel whilst attempting to gain entry, successfully increasing mussel valve gape during entry from 3.7 to 5.5 mm. A pheromone-based mate location system is likely used by this crab to greatly reduce the risks associated with the location of females.

## Abstract (fr)

Les crabes petit pois sont globalement des symbiotes omniprésents dans le milieu marin, qui ont de graves conséquences économiques dans la production par l’aquaculture de plusieurs espèces majeures de bivalves. Cependant, on en sait peu sur leurs interactions hôte-parasite, en particulier le comportement d’accouplement de ces parasites, qui pourraient s’avérer utile pour contrôler leur infestation dans l’aquaculture. Dans cette étude, le comportement de recherche de partenaire sexuel par le crabe mâle, *Nepinnotheres novaezelandiae* (Filhol, 1885), a été observé chez leur hôte préféré, la moule verte d’importance commerciale *Perna canaliculus*. Étant donné le comportement cryptique du crabe mâle, un nouveau système de piégeage a été mis au point pour déterminer si les crabes mâles quittent leurs hôtes moules en réponse à un crabe femelle en amont. La présence de crabes femelles réceptives situées en amont a réussi à attirer 60 % des crabes mâles de leur hôte sur 24 heures. Les observations du comportement nocturne de recherche de partenaire sexuel des crabes mâles ont été faites dans l’obscurité en utilisant des enregistrements vidéo infrarouge. Les mâles ont passé en moyenne 49 min sur des hôtes vides et n’ont jamais quitté une moule contenant un congénère femelle une fois trouvée, passant 200 min en moyenne pour entrer dans la moule. Les crabes mâles ont été souvent observés en train de caresser le bord du manteau de la moule pour essayer d’entrer, augmentant avec succès l’entrebâillement de la moule pendant leur entrée de 3.7 à 5.5 mm. Il est probable qu’un système de localisation de partenaire sexuel à base de phéromones est utilisé par ce crabe pour réduire considérablement les risques associés à la recherche des femelles.

## Introduction

The parasitic New Zealand pea crab, *Nepinnotheres novaezelandiae* (Filhol, 1885) Ahyong & Ng, 2008 [1, 23], has been shown to cause an estimated 30% reduction in the size of commercially aquacultured green-lipped mussels (*Perna canaliculus*), resulting in industry-wide losses in production equivalent to US$ 2.16 million annually [10, 31, 47]. The parasite’s impact is primarily a result of the crab living inside the host mussel and stealing food, as well as eroding the mussel’s gills resulting in stress and decreased rates of filter feeding [10, 47, 51]. *Nepinnotheres novaezelandiae* is found widely throughout New Zealand’s coastal waters, infecting wild populations of green-lipped mussels [29]. Although green-lipped mussels are the preferred host, the parasite can also be found in a wide range of other bivalve species where they act as potential reservoirs capable of rapidly infecting populations of large coastal bivalves such as farmed mussels [37].

Recent research on green-lipped mussel farms has shown that 5.3% of the mussels were infected with this pea crab which would suggest that the location of mates by adult males, which make up only 17.6% of the population, could be particularly challenging because females were distributed evenly throughout the mussel farm [47]. Despite these seemingly poor odds, 89.3% of mature female crabs were found to be gravid with fertilised eggs, indicating a high rate of mating success. Establishment and growth of *N. novaezelandiae* in newly seeded mussel farms from larval settlement is swift as the species appears capable of rapid infection and tremendous reproductive output [49]. For example, a 10-month-old population of farmed green-lipped mussels contained a crab population in which 86.4% were mature females carrying with a mean brood size of 2592 (±579 *SE*) and totalling an estimated 241 million eggs for the farm [46].

Locating and accessing mates provides a potentially dangerous set of circumstances for male *N. novaezelandiae*. Mature male crabs must squeeze out of the gaping valves of their host mussel without being crushed in order to search for female mates [6, 46]. Males of the oyster pea crab, *Pinnotheres ostreum*, were observed to be routinely crushed when attempting to enter or exit an oyster host and although 70% of males were able to make entry into a host, 13% of them were crushed in the process [44]. Once male pea crabs successfully exit their bivalve host, either in a mussel farm or wild mussel bed, they are then exposed to an increased risk of predation since these environments have high diversity and abundance of predatory fish [35, 36]. Despite understanding the challenges faced by male crabs, it is still not understood what cue triggers the males to leave the protection provided by their host and what specific behaviours they use to mitigate the associated risks.

Crushing of mature male crabs and predation are thought to be the likely cause of a female-biased sex ratio in populations of *N. novaezelandiae* where typically about 80% of individuals are female, despite the sex ratio of immature crabs being equal [29, 33, 47]. Therefore, male pea crabs must balance the risk of mortality with the chances of reproductive success through successfully locating a mate [41, 49]. Any means to reduce the risks of mortality whilst locating a mate would be highly beneficial to male fitness and could be expected to be positively selected for. Manipulation and exploitation of natural host behaviour by parasites to minimise the risks associated with reproduction does occur in other species, such as parasitic worms [26, 34, 39, 45, 54], and is often crucial in increasing the breeding success and transmission of parasites to new hosts [15, 34, 38].

One of the potential ways male *N. novaezelandiae* may facilitate the location of mates is through the use of pheromones [46], a method used by many crustaceans and which has been observed in some Pinnotherids [3, 20, 21, 40]. Indeed chemoreception has been highlighted in Pinnotherid species by Ambrosio and Brooks [2] for host recognition [17, 42], habitat selection [5, 24, 43], predator avoidance [11, 16] and conspecific signalling [4, 28]. Chemical cues are thought to be important in adult *N. novaezelandiae* in selecting host bivalves based on host odour but have not been demonstrated for mate location [42]. Therefore, the aim of the current research was to describe the mate-finding behaviour of male *N*. *novaezelandiae*, including interactions with the host green-lipped mussels, and to determine whether there is evidence of pheromone-based location.

## Materials and methods

All crabs and mussels used in experimentation were sourced from a single commercial mussel farm and only host mussels of 9 cm (±0.5) in shell height were used, unless otherwise stated. Crabs and eggs were keyed to developmental stage according to Jones [30] and identified photographically through distinct carapace markings. Crab size represented by carapace width was measured with digital callipers to an accuracy of 0.01 mm. Movement of male crabs between mussel hosts was performed by inserting a gradually sloping stainless steel wedge into the ventral edge of the mussel when the valves were agape. Male crabs were then carefully removed with plastic forceps or encouraged to exit on their own accord by light pressure on one side of the crab towards the open valves. In order to introduce or remove female crabs from a host, the stainless steel wedge was inserted into the gaping valves followed by continual gentle opening pressure between the valves applied with circlip pliers with flattened tips, to spread the force over the shell edge. The stainless steel wedge was then gently pushed between the valves until the desired opening was achieved to remove a female. Where only presence and identification of crabs within hosts was required, this was confirmed visually through the valve gape as this method was less intrusive and stressful for the host mussel and crabs. Movement of crabs was kept to a minimum to reduce stress. Where male crabs exited from their host during experimentation, they were always returned to their original host. Standard errors of means are reported throughout to indicate the reliability of the estimate of the mean.

### Female-induced host exiting by male crabs

**Figure 1.**
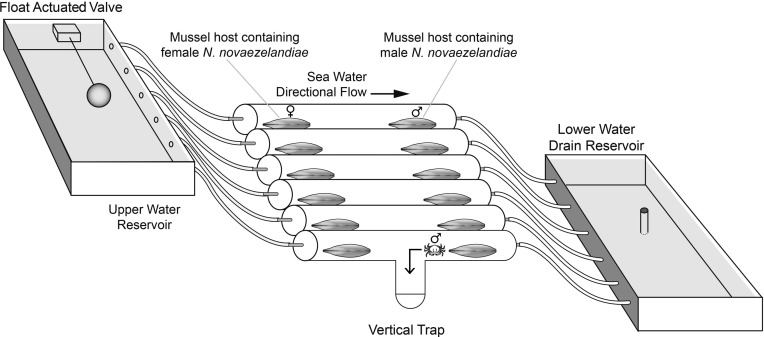
Polyvinyl chloride (PVC) directional flow tubes measuring 35 cm in length and 6.5 cm in diameter with a 15 cm centrally located vertical trap were placed in parallel and supplied with raw seawater to produce a directional flow. A float actuated valve in an upper reservoir provided equal water flow through the tubes at all times. Females were placed upstream and males downstream within their respective hosts. Male crabs exiting their hosts and attempting to proceed towards the female’s host fell into the vertical trap and were unable to exit. A common drain reservoir collected outflowing water.

An experiment was carried out to determine whether female crabs could elicit male crabs to exit their host mussel downstream, as an indicator of the possible use of a pheromone cue. A series of 10 polyvinyl chloride (PVC) tubes measuring 35 cm in length and 6.5 cm in diameter, each with a 15 cm long vertical trap located half way along the tube, were placed in parallel and supplied with raw seawater to produce a directional flow 0.5–1.0 cm s^−1^ confirmed by tracing plumes of released food colouring (Fig. 1). A female crab within its original mussel host was placed in the upstream section of the tube and a male crab in its mussel host in the downstream section of the tube. Vertical PVC rods at the end of the female and beginning of the male section prevented the host mussels from moving across the trap with byssus thread walking. The bottom section of the vertical trap was removed daily to check for the presence of male crabs that had attempted to move towards the mussel containing the female crab. Nine of the tubes contained hosts in the upstream section bearing mature females with early stage, late stage or no eggs and one tube contained an empty host as a control. Two experiments were conducted. The first experiment ran for 30 days and over this time any males found in traps at every 24 h census were returned to their original host mussel and monitored for the occurrence of repeated host exiting behaviour when exposed to the same female. The position of the control tube containing the mussel that did not host a female crab was randomised after each census to ensure movement was not influenced by the tube and a new control mussel substituted. In total, 30 mussels without a female crab were tested against the control male to rule out exiting behaviour in response solely to the host. In the second experiment, all 10 males (including the male crab used in the previous control treatment) were exposed to a single female known to elicit a male response for a 24 h period, each consecutively in the same tube.

### Host entrance behaviour by male crabs

Infrared recording methods were used to register male crabs gaining entry to mussel hosts of female crabs in the absence of visible light. Video recordings were captured of crabs placed in a transparent polycarbonate tube measuring 35 cm in length and 6.5 cm in diameter. Within the tube, a male crab was placed about 10 cm downstream of a female crab, each housed within a host mussel, and the behaviour of the male crab recorded continually with a Sony HDV (HVRA1 U) camera under infrared illumination. A continuous directional flow (female to male crab) of raw seawater of 0.5–1 cm s^−1^ was maintained in the tube during recordings. Recordings were taken in 24 h segments for each crab with a total of five different pairs of male and female crabs and their hosts observed. Video recordings were analysed to determine the following aspects of the behaviour for each male crab: attempts to enter host, time spent at entry point of mussel (inhalant siphon), total time spent on female crab’s host mussel and total time from exit of male’s host mussel to successful entrance of female’s mussel. Individual attempts to enter a host by the male crab were defined as the mussel closing and the male crab withdrawing from between the gaping valves to avoid being crushed.

To determine whether male crab behaviour causes an increase in the valve gape of a mussel to facilitate access to the female crab contained inside, measurements using image analysis software were taken from still images sourced from the video recordings. Repeated measurements (*n* = 10) from the same location of the normal valve gape of each mussel at the mussel’s anterior ventral opening from a random selection of instances and the valve gape during male crab entrance (*n* = 10) were made for each of the five different pairs of male and female crabs and their hosts that were observed. Male crabs used in these experiments (*n* = 28) were measured for their carapace height with digital callipers to an accuracy of 0.01 mm. The mean measurements for normal mussel valve gape in the absence of a male crab and the valve gape of the same mussel at the time of a male crab entering between the valves were compared with a paired Student’s *t*-test analysis after ensuring the data dispersion conformed to underlying assumptions for parametric tests. Even though sample sizes were relatively small, the Student’s *t*-test is known to remain robust [18, 22].

### Mussel valve gape behaviour

Experimental data suggested that pea crabs display a behaviour of stroking mussels into which they were attempting to gain entrance (see Results). We further conducted an experiment to determine whether crabs’ stroking behaviour had the ability to increase the valve gape of mussels, and whether there were diurnal/nocturnal differences in the sensitivity of the host mussels. Two hundred mussels were placed in a 40 × 60 × 12 cm aquarium with constant inflow of filtered (10 μm) seawater and allowed to acclimate for 5 days in ambient day:night conditions. The daytime (12:00 h) and nighttime (24:00 h) treatment consisted of gently stroking the mantle edge of 30 randomly selected mussels from the holding tank of 200 mussels for 10 min with a pair of metal forceps, in a manner similar to the behaviour of male pea crabs and recording the observed responses of the mussel subjects.

### Mussel valve gape profile

**Figure 2.**
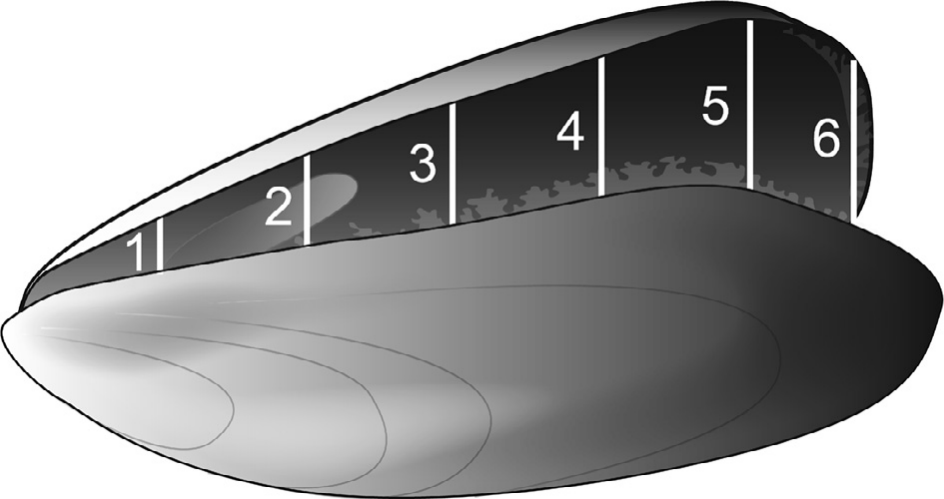
*Perna canaliculus* valve gape profile measurement locations along the ventral margin of the shell when the mussel was open on the shell hinge. Predetermined locations included: the common entry location for male crabs (point 5), the distance between this point and the umbo equally divided (points 1, 2, 3, 4) and the anterior edge (point 6).

To ascertain the optimal location of entry for *N. novaezelandiae* represented by the site of greatest gape of the valves, a profile of the valve gape around the exterior margin of the valves of *P*. *canaliculus* individuals was measured. Mussels (*n* = 100) between 89 and 91 mm in shell height were measured at six locations along the ventral and anterior edges of the valves with a digital calliper to an accuracy of 0.01 mm (Fig. 2). The mussels were held open on their hinge for measurement by insertion of a 7.7 mm stainless steel wedge at the shell apex (Fig. 2 – point 3). The locations for measuring the valve gape were selected based on being the common entry location for male crabs (point 5), the distance between this point and the umbo equally divided (points 1, 2, 3, 4) and the anterior edge (point 6).

### Male crab mate location behaviour

**Figure 3.**
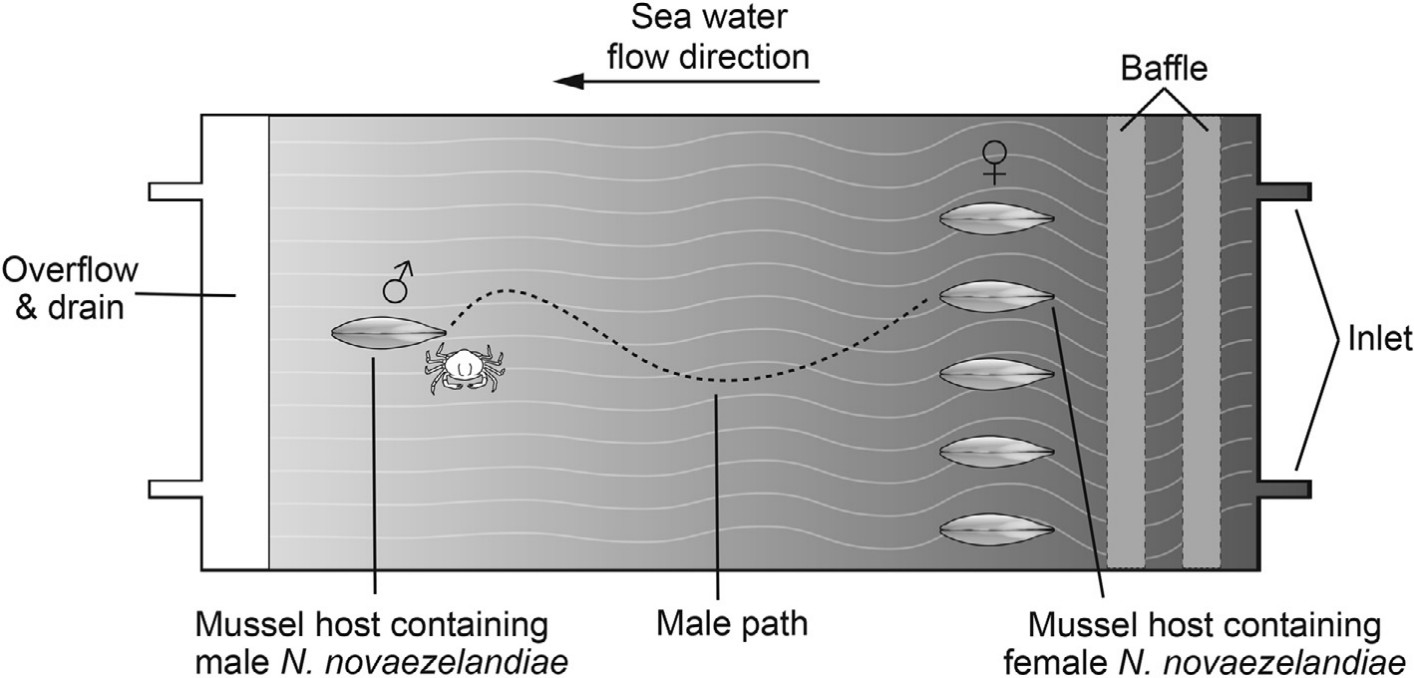
Behavioural observation chamber with five *P. canaliculus* hosts upstream in laminar flow seawater, one containing a female *N. novaezelandiae* that is randomly positioned within the row. A single host with a male was fixed downstream in the chamber and its movements were recorded in darkness with infrared lighting and video recording.

**Figure 4.**
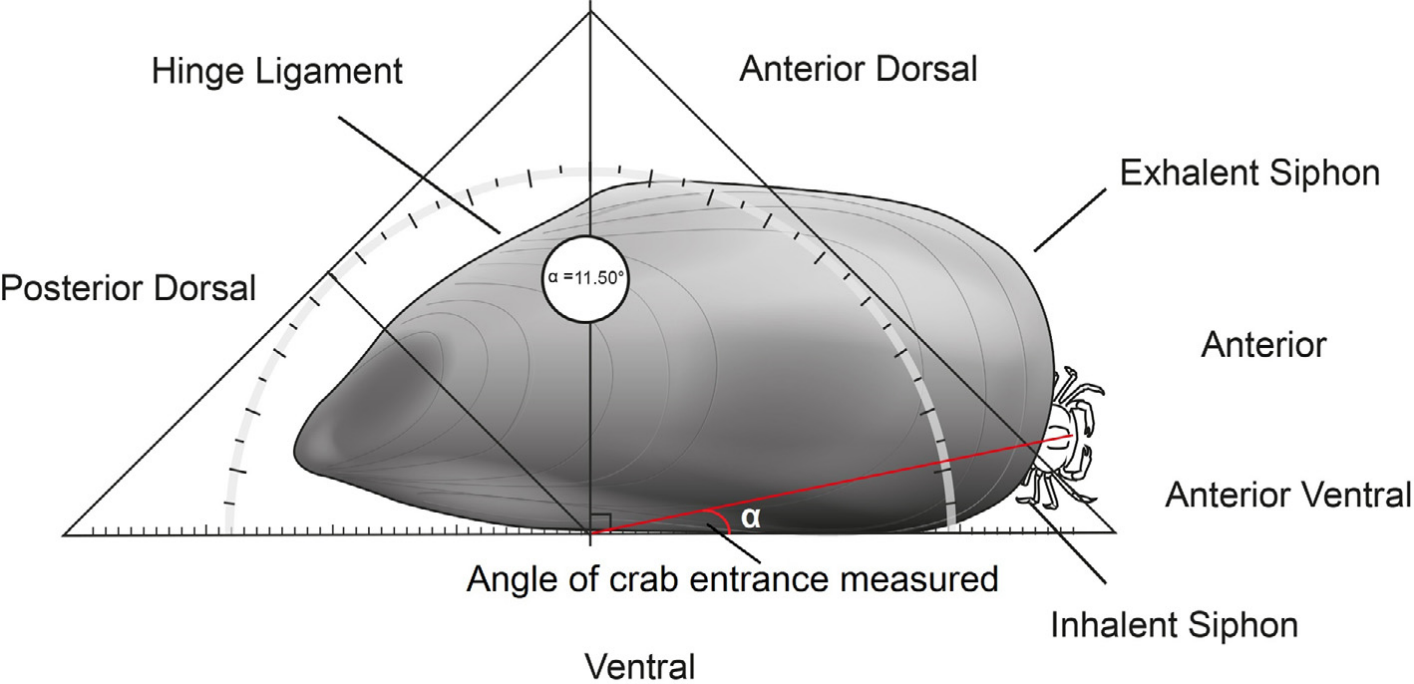
The host entry location of male *N. novaezelandiae* was determined as an angle from the point at which a perpendicular line from the anterior dorsal end of the shell hinge ligament bisects a line running along the ventral edge of the *P. canaliculus* host (*α*).

Observations were made of the behaviour of male crabs in locating a female housed in a mussel host when offered a choice among five mussels of which only one contained a female crab. The observations were undertaken in a tank of 44 × 54 × 13 cm using filtered (10 μm) seawater which entered through a series of collimators before reaching the main chamber where laminar flow between 1 and 2 cm s^−1^ was confirmed prior to experimentation (Fig. 3). A mussel containing a single male crab was fixed downstream by a cable tie and five mussels were placed about 18 cm upstream, 7 cm apart across the width of the laminar flow tank with the umbo of the mussel facing downstream. One of the five mussels upstream was randomly selected to contain a female crab. The tank was held in an absence of visible light but under illumination with an infrared light source and male crab behaviour was recorded with a Sony HDV (HVRA1 U). The experiment was run for a maximum of five days or until the male had located and entered the female crab’s host mussel for each individual male crab. Observations were made of the behaviour of 10 male crabs. Two identical connected chambers were located next to each other with one chamber run as a control and one for experimentation. The control experiment consisting of five empty hosts with a male downstream was run simultaneously with experiments under exactly the same conditions. Video recordings were analysed to determine the following aspects of the behaviour for each male crab: number of mussels visited, time spent on mussels with and without a female crab inside, total time to locate mussel hosting female crab and total time from exiting male host to entering female’s host mussel. To determine whether the choice of mussels to investigate by male crabs was at random or directed, the number of mussels visited before a male crab successfully located the female in the laminar flow choice chamber was analysed by a Chi-square test of goodness of fit. The mean amount of time that male crabs spent on empty hosts compared to the female’s host was analysed with Student’s *t*-test after ensuring the data dispersion conformed to underlying assumptions for parametric tests. In addition, the location around the shell margin of the mussel at which male crabs entered the mussel was determined from still images (*n* = 10 images) taken from the video recording at the moment of male crab’s entry into the mussel host of the female crab. The position of entry on the mussel valves by the male crabs was measured with Markus-Bader digital ruler measuring software as an angular value from the intersection of a line drawn from the edge of the hinge ligament to the anterior dorsal edge and ventral edge of the mussel valves (Fig. 4).

## Results

### Female-induced host exiting by male crabs

In the first 30-day experiment, the male crabs in the control treatment were never observed outside of their host. Four of the nine female crabs caused their respective male crabs to exit their hosts and fall into the vertical trap (Table 1). In the second experiment, the female crab previously found to elicit host-exiting behaviour was found to successfully elicit exiting behaviour in a further five different male crabs, as well as a male crab which had previously been used in the control treatment, confirming that it was responsive to attraction cues from females when present (Table 1).

**Table 1.** Male exiting behaviour in response to Stage V females both with and without eggs of various development stages being present (Experiment 1). Exiting response over a 24-h period of the same male crabs to a female crab known to have previously elicited male exiting.

Male number	Female stage	Egg stage	Occurence of male in trap	Elicited by female
			Experiment 1	Experiment 2
1	Control	–	0	✓
2	Stage V	Early	0	✕
3	Stage V	Early	0	✕
4	Stage V	Early	0	✓
5	Stage V	Late	0	✕
6	Stage V	Late	1	✕
7	Stage V	Late	0	✓
8	Stage V	None	4	✓
9	Stage V	None	2	✓
10	Stage V	None	4	✓

### Host entrance behaviour by male crabs

The pattern of behaviour of male *N. novaezelandiae* entering a female crab’s mussel host was consistent among all five individuals observed. After making initial contact with the mussel containing the female, the male crab would proceed directly to the anterior region of the mussel and inspect the area near the exhalent and inhalant water plumes. The male crab would then remain in this region until access was gained into the mussel (Supplemental material video 1 – Male Pea Crab Entrance of Host Mussel). The crab would often position itself with its 4th pair of legs in the valve gape once the mussel valves were slightly agape. In this position it would stroke the mussel’s mantle edge with various pairs of legs, typically causing the mussel’s mantle rim to undulate in response. This stroking behaviour would continue on and off until the mussel’s valve gape was sufficient for the crab to enter. Stroking of the mussel mantle was present for three of the observations (*n* = 5) (Supplemental material video 2 – Male Pea Crab Stroking Host Mussel). Male crabs took on average 3.4 (±0.8) attempts to enter the host before succeeding (*n* = 5). The mean time spent at the inhalant siphon awaiting the appropriate conditions for entry to the host was 184 (±63) min (*n* = 5). The mean total time spent on the female’s host at the siphon and investigating the mussel was 352 (±195) min (*n* = 5). On average it took 413 (±185) min from the male’s initial exit of his host through to accessing the female inside her mussel host, including time searching for the female host mussel, time spent on the female’s host and entering the mussel (*n* = 5). The shortest time period from the exit of the male crab’s host and subsequent entrance of the female crab’s host was 91 min and longest was 1101 min.

**Figure 5.**
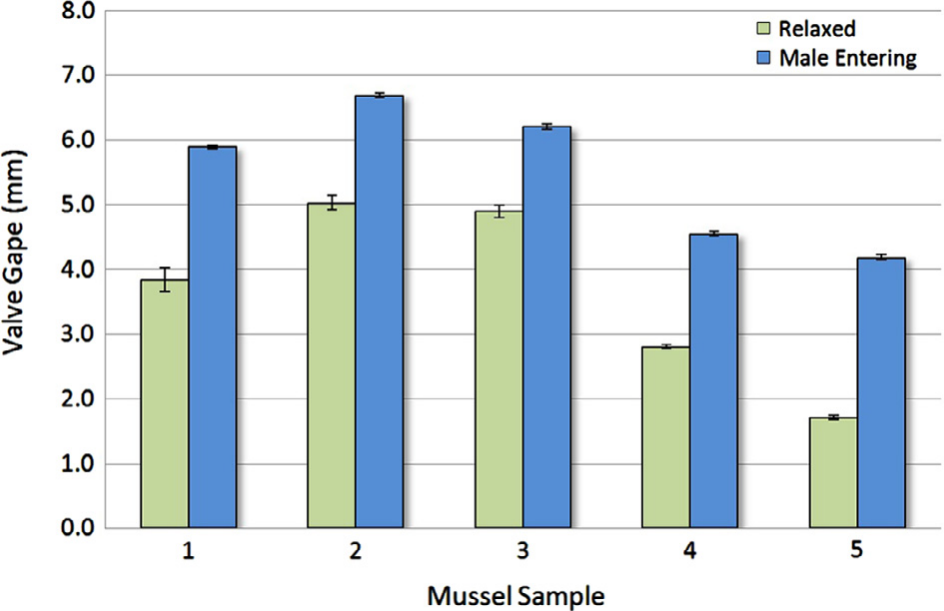
Mean normal valve gape compared with the maximum valve gape of five different female *N. novaezelandiae* hosts during male crab entrance. The combined mean normal valve gape of 3.6 mm (±0.63 *SE*) differed significantly (Student’s *t*-test, *P* < 0.001) from the mean valve gape during male crab entrance 5.5 mm (±0.48 *SE*).

The mean values for valve gape of mussels with and without a male crab attempting to enter the mussel differed significantly (Paired Student’s *t*-test, *P* < 0.001) at 5.5 (±0.48) and 3.6 (±0.63) mm, respectively (Fig. 5). The mean height of male crabs used in experimentation (*n* = 28) was 4.6 (±0.7) mm and ranged from 3.8 to 5.1 mm, which was consistent with the valve gape measured in the mussels at the time of entry by the male crab.

### Mussel valve gape behaviour

During the daytime treatment (12:00 h), all 30 mussels when gently stroked along the mantle edge immediately closed on the forceps with enough force to require them to be pried out whilst holding the mussel down. In the nighttime treatment (24:00 h), 22 of the 30 mussels allowed continuous stroking along the mantle edge over the 10-min period and the entire 4 cm of the forceps to be inserted into the mantle cavity, however, no change in valve gape was observed. Of the remaining eight mussels, three allowed access at first but closed within the 10-min period, and five closed shortly after contact of the forceps with the mantle (Supplemental material video 3 – Diurnal Relaxed Host Mussel).

### Mussel valve gape profile

**Figure 6.**
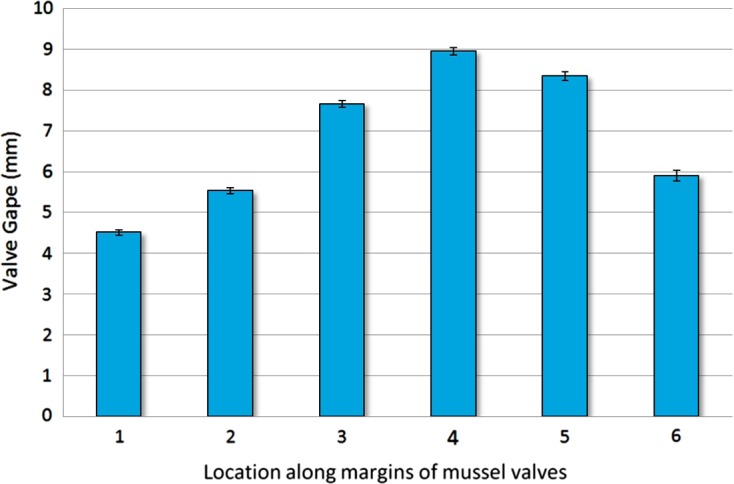
Mean valve gape of host *P. canaliculus* at a range of measurement locations along the ventral margin of the animal under normal feeding conditions (*n* = 100). The widest mean valve gape of 8.95 mm (±0.093 *SE*) was located at point 4 at the middle of the inhalant siphon.

The valve gape of green-lipped mussels gradually increased from 4.5 (±0.06) mm at point 1 near the umbo on the ventral edge through to point 4 where the maximum valve gape mean of 8.95 (±0.09) mm was observed (Figs. 2 and 6). The mean gape then decreased on the anterior edge to 5.95 (±0.13) mm. Points 4 and 5, where the maximum valve gap was observed, were also the identified region where male crabs consistently choose to enter and exit the mussel hosts.

### Male crab mate location behaviour

**Figure 7.**
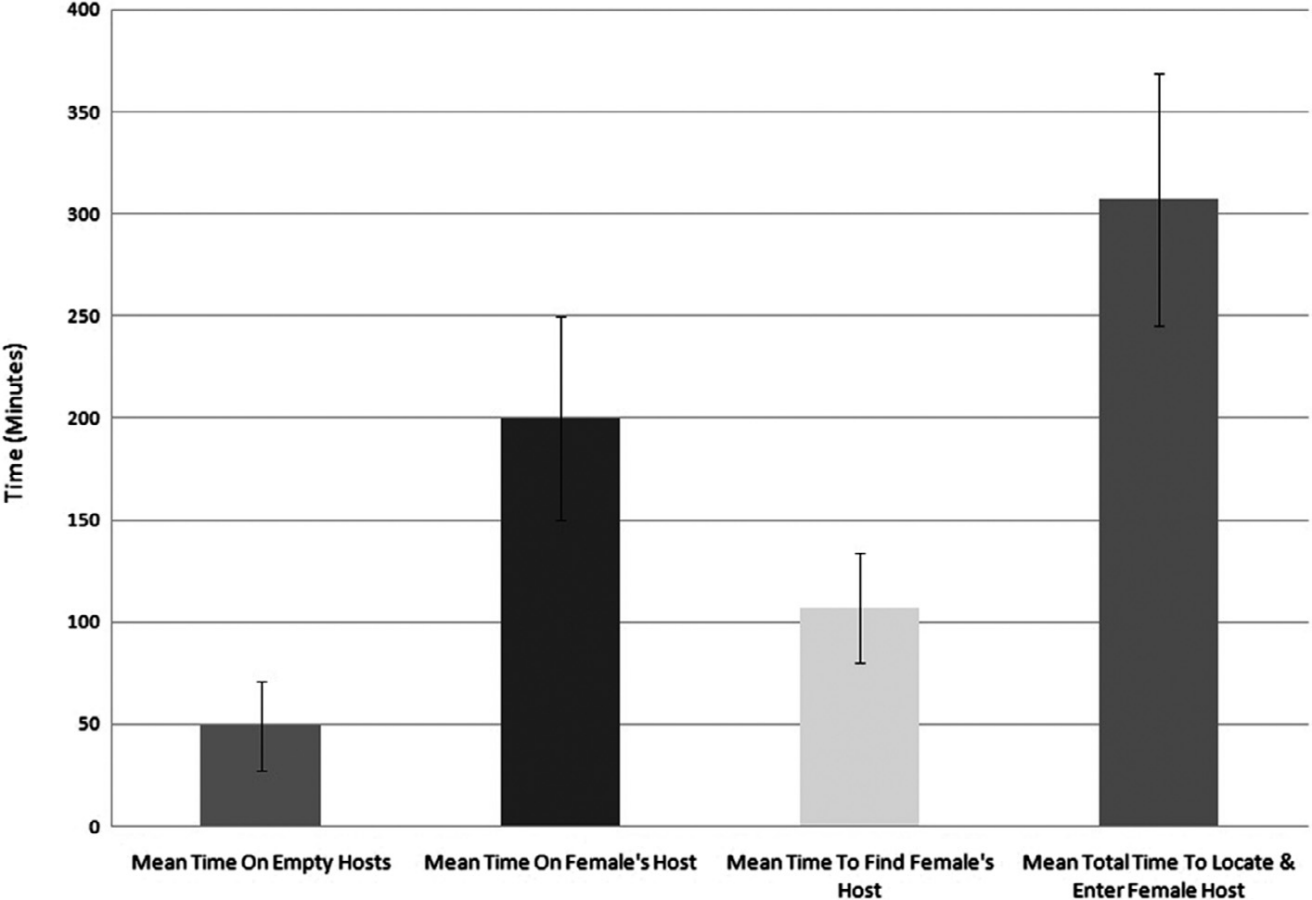
Time data on male searching behaviour indicated that the amount of time male crabs spent on mussels that did not host a female crab was 49 (± 22 *SE*) min, which was significantly less (Students *t*-test, *P* = 0.025) than the mean amount of time they spent on hosts containing a female (200 ± 50 *SE* min).

Males leaving their host in search of mates visited on average 2.6 (±0.8) empty mussels before locating the single mussel among the five hosting a female crab. Only a single incidence of a crab proceeding directly to the mussel hosting the female crab was recorded and male crabs were never observed returning to the same mussel without a crab once it had been inspected. The mean amount of time males spent on mussels that did not host a female crab was 49 (±22) min, which was significantly less (Student’s *t*-test, *P* = 0.025) than the mean amount of time they spent on hosts containing a female (200 ± 50 min) (Fig. 7). Whilst visiting potential host mussels and exploring the laminar flow chamber, male crabs took on average 107 (±27) min to find the female crab’s host mussel and a total mean time of 307 (±62) min from leaving their host to entering the mussel hosting the female crab (Fig. 7). The number of hosts a male crab visited before locating the mussel hosting the female crab was less than expected by chance alone (Chi-square analysis, χ^2^_4_ = 9.49, *P* < 0.05, *n* = 10) and in the approx. 40 days of recording the randomly selected males in the connected flow chamber were never observed outside their hosts. Male crabs (*n* = 10) always entered a host through the valve gape at the inhalant siphon and the mean angle of entrance on the mussel valves hosting the female crab was 11.5° (±3.3°) (Fig. 4).

## Discussion

Male *N. novaezelandiae* must balance the benefit of mating versus the predation risk from venturing out from the protection offered by their mussel host and the risk of being crushed by a mussel whilst entering or exiting [32, 33]. These risks are significant: for example, 13% of male crabs of *P*. *ostreum* have been found to be crushed to death during any entry or exit event of their oyster host [44]. The results of the current study suggest the male crabs have behaviour that may help to minimise these risks and may be applicable to the predictive behavioural model of Baeza and Thiel similar to other crab species [8]. For example, in this study male crabs were only observed to exit their hosts when in darkness, which was consistent with a lack of success by the authors to elicit any response from male crabs in ambient light conditions during previous experiments. *Perna canaliculus* hosts were hypersensitive to their mantle rim being touched during the day and all mussels closed firmly upon contact of the mantle edge with the forceps. The same behaviour was not observed at night when 73% of experimental mussels allowed gentle stroking of the mantle edge with forceps, suggesting that mussels were more vulnerable to entry by the parasitic crabs at night, a behaviour that concurs with the timing of mate searching activity by male crabs. Nocturnal behaviour would also reduce the predation risk to male crabs from mobile visual predators, such as fish, further reducing the associated risks whilst searching for a mate, as has been observed in other nocturnally active crustacean symbiont species [14, 25, 50, 52].

The first step in locating a mate for a male crab is exiting its host, the timing of which is mediated by the mussel itself. Once a male exits its host mussel, the amount of time spent outside of the protection of a host searching for a female would potentially greatly influence their risk of predation. This risk could be expected to be reduced if those female crabs that were receptive to mating released a cue at night to attract males and/or confirm the female was present within a host mussel, without the need for the male to first enter every available mussel to search for the presence of a mate. When offered a choice of five mussels in our behavioural arena, of which only one contained a female crab, the male crabs showed a significant preference for selecting mussels containing females (*P* < 0.05), and they never returned to re-inspect mussels without a female, never attempted to enter a mussel that did not host a female crab and never abandoned a mussel hosting a female crab once found. The mean time spent on empty hosts was 49 (±22) min which is significantly less (*P* = 0.025) than the time spent on the host mussels containing a female crab (200 ± 50 min). In this same experiment, the control crab located in the adjacent chamber was never observed outside of its host in the approx. 40 days of recordings. The lack of male exiting in the control eliminates the chance of a signal coming through the inflowing water. Also, since the system was attached and recordings and movements of crabs occurred only in complete darkness, this strongly suggests that acoustic or visual cues are not the main source of mate attraction, although this would need to be further examined before being excluded.

In crustaceans, mate searching can depend on different types of cues including visual, chemical and acoustic, although chemical cues are more widely reported among aquatic species, especially crustaceans [4, 12, 20, 27, 40]. Our results suggest that mate location in *N. novaezelandiae* is not random but directed by a cue that is available to male crabs outside of the female crab’s host mussel. Individuals of many species of crustaceans are separated over various distances and need to locate each other for mating [19]. Some crab species are known to use acoustic or vibratory cues to attract mates. However, no evidence of this was observed during our experiments [40]. Waterborne chemical signals have the potential to be effective over a significant distance and have been observed to be emitted by both males and females in a variety of crustacean species [5, 7, 13, 17, 20]. Our results suggest a pheromone produced by female *N. novaezelandiae* elicits male crabs to exit their hosts and assists them in locating the female crab.

Of the nine pairs of male:female crabs employed in the experiment using the PVC tubes, four out of nine male crabs responded to their matching female crab and three of these male crabs showed the same response repeatedly over a 30-day period. This result suggests that the five female crabs that did not elicit movement from male crabs may not have been producing a mating cue. In contrast, when a female crab that had previously elicited a male crab to repeatedly exit his host mussel was tested individually with 10 different male crabs, 60% of the males exited their hosts within 24 h. This percentage could have been higher but exiting of the hosts is mediated by the male’s host opening and if this did not occur the male could not have exited. These results support the theory of a chemical cue although the use of a visual or acoustic cue, or combination of cues, would need to be verified further before definitively associating the mate attraction of this species. However, the ability of female crabs to remotely attract mates may be particularly important in this species in which the proportion of mature male crabs in a population can be relatively low (17.6%) and the number of mussels hosting a female is low (typically <10%) both of which would otherwise limit the successful location of mates by males [46].

The behavioural observation chamber, directional pheromone tubes and mate access recordings were all performed with stage V females. Traditionally, pea crabs copulate when both sexes are hard stage earlier on in the developmental cycle. This research suggests a potential pheromone attraction system involving stage V females that is capable of eliciting a response from mature male crabs. The male attraction response observed in this research suggests that copulation in *N. novaezelandiae* may be able to occur with mature females. Post-hard stage mating has not been observed in other species of pea crab.

Male *N. novaezelandiae* always carefully positioned themselves and entered the mussel host of the female crab by passing through the inhalant siphon at a specific area of around 11.5° (±3.8°) (Fig. 4). The valve gape in this region (points 4 and 5, Fig. 6) is the widest and would help facilitate entry for the crabs. Once the male crab is located at the inhalant siphon, the crabs reverse themselves against the margin of the valves at the inhalant siphon with their 4th pair of legs inserted into the mussel mantle. In this orientation, males were observed stroking the mussel’s mantle edge with their legs while waiting for the valve gape to open sufficiently to allow the crab to enter the mussel. Our attempts to mimic this mantle stroking behaviour with forceps did not increase the valve gape of the mussel, but this may have been due to the limited time period (10 min) of experimentation compared to the extended time that more patient male crabs performed this action (mean = 200 ± 50 min, *n* = 10).

Mean male crab height (4.6 ± 0.7 mm) was larger than the mean valve gape of mussels (3.6 ± 0.63 mm) under normal conditions. Therefore, in order for the male crab to make entrance to the mussel host, the valve gape would need to exceed this value and mantle stroking may be involved (Fig. 5). When making entry to the female’s mussel host compared to the normal valve gape position, there was a significant (Students *t*-test, *P* < 0.001) increase of 36% in valve gape (Fig. 5). This suggests that there may be an interaction between the male crab and the female crab’s mussel host to facilitate the entrance of the male. However, it remains to be determined whether this response by the mussel is initiated by the male, female or both.

## Conclusions

This study suggests that female *N. novaezelandiae* use a pheromone to attract mates and that once cued, male crabs follow consistent behavioural patterns associated with the exit of their own host and the subsequent entry of the female crab’s host. Research to further confirm the presence and identify the specific chemical composition of a potential pheromone is needed and could be based upon a similar behavioural bioassay used in this research. The chemical signalling, behavioural patterns and potential host manipulation by male crabs have evolved to increase mating success and decrease male mortality because mate acquisition by females appears to be a potentially limiting factor to breeding success in this species. However, the use of an artificial pheromone to attract males provides a potentially viable avenue to reduce infection levels with this commercially significant parasite in aquaculture.

## Supplemental material

**Video 1. Male Pea Crab Entrance of Host Mussel.** The behaviour of a male *N*. *novaezelandiae* entering a green-lipped mussel, *P. canaliculus*, hosting a female crab by reversing themselves against the margin of the valves at the inhalant siphon of the mussel. Recorded in darkness using infrared lighting.

**Video 2. Male Pea Crab Stroking Host Mussel.** The behaviour of a male *N*. *novaezelandiae* attempting to gain entrance to a green-lipped mussel, *P. canaliculus*, hosting a female crab by stroking the mantle of the mussel with the tips of the legs. Recorded in darkness using infrared lighting.

**Video 3. Nocturnal Relaxed Host Mussel.** Demonstration of the nocturnally relaxed state of the valves of the green-lipped mussel, *P. canaliculus*, by stroking the sensitive mantle of the mussel with metal tweezers, which if conducted during daytime would cause immediate closure of the valves. Recorded in darkness using infrared lighting.
